# Photocontrolled Dopamine Polymerization on DNA Origami with Nanometer Resolution

**DOI:** 10.1002/anie.201911249

**Published:** 2019-12-27

**Authors:** Pia Winterwerber, Sean Harvey, David Y. W. Ng, Tanja Weil

**Affiliations:** ^1^ Max Planck Institute for Polymer Research Ackermannweg 10 55128 Mainz Germany; ^2^ Institute of Inorganic Chemistry I Ulm University Albert-Einstein-Allee 1 89081 Ulm Germany

**Keywords:** DNA nanotubes, DNA origami, photopolymerization, polydopamine, stability in water

## Abstract

Temporal and spatial control over polydopamine formation on the nanoscale can be achieved by installing an irradiation‐sensitive polymerization system on DNA origami. Precisely distributed G‐quadruplex structures on the DNA template serve as anchors for embedding the photosensitizer protoporphyrin IX, which—upon irradiation with visible light—induces the multistep oxidation of dopamine to polydopamine, producing polymeric structures on designated areas within the origami framework. The photochemical polymerization process allows exclusive control over polydopamine layer formation through the simple on/off switching of the light source. The obtained polymer–DNA hybrid material shows significantly enhanced stability, paving the way for biomedical and chemical applications that are typically not possible owing to the sensitivity of DNA.

## Graphical abstract

**When dopamine is photopolymerized** on DNA origami, precisely patterned nanoobjects are obtained with unprecedented spatiotemporal control and nanometer resolution. This approach combines the advantages of visible light as an external stimulus and the unique addressability of the DNA origami methodology.

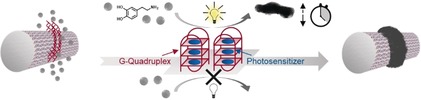

The creation of functional materials with high control and precision during the synthesis process to provide a shape‐customized product is one of the major objectives in the field of nanoscience. Inspired by Nature, bottom‐up strategies often exploit the self‐assembly capacity of various building blocks to produce ordered structures such as liposomes, polymeric nanoparticles, and viral protein mimics.^1^ Although these structures exhibit a relatively high level of uniformity in terms of size, shape, and functionality, they often lack diverse shapes and the potential for asymmetric and orthogonal molecular modifications.^2^ DNA origami technology, however, provides the opportunity to simultaneously incorporate chemically diverse functional components with controlled stoichiometry to design nanoobjects with ultimate molecular control.^3^

Rationally designed DNA origami objects originate from the sequence‐specific binding properties of DNA: a long, single‐stranded DNA scaffold is folded into a distinct architecture guided by a set of short staple strands, which hybridize at programmed positions along this scaffold. Each staple strand can serve as an attachment point for modifications, either directly attached to the staple strand or through a complementary oligonucleotide, furnishing an unprecedented combinatorial platform for designing versatile nanoobjects.^4^ The molecular positioning of, e.g., chromophores, nanoparticles, and drug molecules on the origami surface renders these nanoobjects versatile research tools for various applications in biophysics, medicine, and engineering.^5^ Super‐resolution microscopy employs dye‐modified origami as a nanoscopic ruler for calibration and applies DNA‐PAINT to in vitro applications, making use of transient binding events to monitor target–probe interactions.^6^ Here, fluorescence allows Förster resonance energy transfer (FRET) studies, which are extremely sensitive to slight shifts in the distance between donors and acceptors, thus making it a powerful tool to monitor changes in the conformation of the DNA construct.^7^ In addition, programmable arrays of antenna systems on DNA platforms provide exciting insights into light‐harvesting complexes and energy transduction efficacy.^8^ The precise positioning of nanoparticles, e.g., gold nanoparticles and ‐rods or nanodiamonds on DNA structures opens the possibility to investigate near‐field plasmonic coupling with respect to distance, chirality, and orientation.^9^ Furthermore, biological applications benefit from DNA origami's advantages in the study of enzymatic nanoreactors, in which the spatial relationship of reaction cascades of enzymes can be examined.^10^ The clustering behavior of cell‐surface receptors can be studied by ligands organized with nanoscale precision on DNA origami.^11^

However, the prospects of DNA origami methodology go beyond simply positioning objects and may be expanded to perform chemical reactions or even the bottom‐up synthesis of macromolecules. In this respect, in situ atom‐transfer radical polymerization (ATRP) on DNA origami is suitable for the fabrication of polymers with precisely designed nanopatterns.^12^ By applying initiator sites mimicking horseradish peroxidase activity, it is possible to create template‐mediated polyaniline and polydopamine structures of various shapes on the nanoscale.^13^ However, all these reactions are chemically triggered, which does not provide real‐time control beyond that of stoichiometry. As such, the use of photochemistry offers an attractive strategy to exert temporal control.^14^ The spatial resolution to create nanopatterns using photochemistry is typically limited by the wavelength of light. Therefore, we envision that these limitations could be overcome by the combination of photochemistry and DNA origami.

Herein, we have developed a photopolymerization reaction using a guanine‐rich quadruplex (G4) with the embedded photosensitizer protoporphyrin IX (PPIX). The production of reactive oxygen species (ROS) upon irradiation with white light was used to initiate the oxidative polymerization of dopamine with spatiotemporal control (Figure 1 A). These G4 sequences, which act as reaction centers for dopamine, were arranged in designated patterns on the DNA origami surface. The array of reaction centers caused dopamine to polymerize and, due to the adhesiveness of the oligomers, allowed the polymer to be imprinted on top of the G4 pattern. With this approach, we have bridged the fields of DNA origami technology and photoinitiated polymerization to create precisely templated nanostructures far smaller than the wavelength of light.

**Figure 1:**
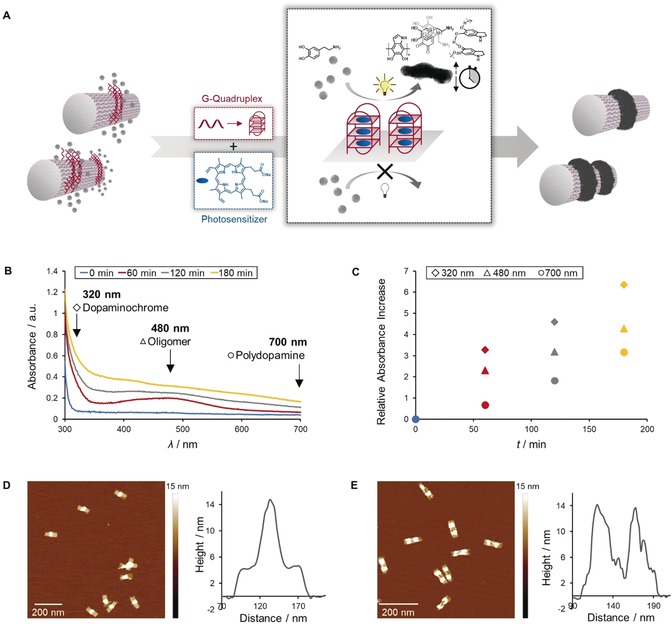
A) Concept of light‐triggered polydopamine formation on DNA origami. Upon the irradiation with visible light, the photosensitizer (protoporphyrin IX), which is embedded in G‐quadruplex (G4) structures on the DNA template, locally catalyzes the oxidation of dopamine to polydopamine, resulting in well‐defined polydopamine–DNA hybrid materials with nanoscale dimensions. B,C) The multistep polymerization of dopamine to polydopamine can be followed by recording the absorbance of the intermediates and final product at 320 nm (dopaminochrome), 480 nm (oligomer), and 700 nm (polydopamine), which steadily increase. D,E) AFM topographic images reveal a significant increase in height where polydopamine is imprinted on top of the G4 pattern.

The DNA origami platforms—tube I and tube II with one and two rings of reaction centers, respectively—were synthesized from scaffold DNA, staples, and G4‐extended staple strands in a one‐step process, and their integrity was confirmed by atomic force microscopy (AFM), whereby the moderately bulky structure of the G4 sequences appeared as slight dots and lines on the tubes (Figure S2). Steric hindrance due to the curvature guided the folding process towards tubes that present G4 strands on the outside. These extended structures were then exploited as anchors for retaining the photosensitizer protoporphyrin IX at distinct locations within the DNA framework. Upon binding to the G‐quadruplexes, the fluorescence of PPIX significantly increased, indicating successful host–guest binding (Figure S3).^15^ The specificity of this binding event was demonstrated by comparing the employed G4 sequence to an alternately organized guanine‐rich oligonucleotide (Figure S4). To induce the polymerization of dopamine, an oxidative environment was required.^16^ The structure of polydopamine is many‐faceted and still under discussion: Apart from covalent bonds, also noncovalent interactions such as hydrogen bonding and π–π stacking, among others, are present (Figure S5).^16^, ^17^ Common protocols, i.e., immersing a substrate in an aqueous alkaline solution of dopamine, suffered from poor control over polymerization kinetics and spatial resolution.^16^ To suppress the spontaneous self‐polymerization of dopamine, the pH had to be adjusted to the acidic range.^18^ However, in acidic medium, polymerization is inducible only in the presence of strong oxidants such as ammonium persulfate and sodium periodate.^19^ Levkin and co‐workers have shown the first example of a light‐triggered dopamine polymerization using UV light to generate reactive oxygen species (ROS) in situ that act as oxidants.^19^ However, the use of UV light in DNA origami can induce cross‐linking and promote nonlocalized polymerization.^20^ Therefore, we have developed a photosensitizer‐mediated system that can be activated by exposure to visible light.

Protoporphyrin IX is utilized in photodynamic therapy^21^ and we examined its capability to trigger ROS formation, in particular the generation of singlet oxygen (Figure S6). Singlet oxygen generated by irradiation with visible light reacts with imidazole and bleaches *n*,*n*‐dimethyl‐4‐nitrosoaniline (RNO) through oxidation, while no reaction occurs in the absence of light.^22^ The decrease in RNO absorbance by approximately 20 % proved PPIX to be a moderate photosensitizer with rather slow kinetics, facilitating control over the reaction. Furthermore, to control the polydopamine formation, only a narrow pH range proved to permit both initiating and promoting the polymerization process, with pH 6.5 being the most selective (Figure S7). According to our studies, polydopamine formation occurred independently of any external stimulus in neutral and alkaline media. Under acidic conditions, a pH value of 6 allowed the oxidation of dopamine but prohibited subsequent polymerization steps, whereas at pH 5, all reaction was inhibited. At pH 6.5, polydopamine was formed only in the presence of light and photosensitizer, which was crucial for establishing a controlled polymerization process (Figures S8 and S9).

Upon the light‐triggered ROS generation by PPIX and subsequent dopamine oxidation, reaction progress was monitored by recording UV/Vis spectra, tracking the increase of oxidized species (dopaminochrome), precursor oligomers, and polydopamine (Figure 1 B).13b Initially, oxidation of dopamine to dopaminochrome and ongoing oligomer formation was observed, followed by the emergence of polymeric components. To further prove that polydopamine formation is an ROS‐mediated process, we studied the impact of oxygen and imidazole as a singlet oxygen scavenger on the reaction kinetics. Therefore, the reaction was performed according to an alternative protocol in which the system is degassed. Both UV/Vis spectroscopy and AFM imaging revealed a suppression of the polymerization when no oxygen was present (Figures S10 and S11). The effect was even stronger in the presence of the scavenger.

As a first indication of successful polymerization, polydopamine‐coated structures did not show any migration during agarose gel electrophoresis in contrast to unmodified DNA origami (Figure S12). To directly confirm the presence of the polymer and verify its position, a topographical map of the obtained structures was recorded via AFM measurements (compare Figure S2 and Figure 1 D,E). The height profile of both structures exhibited an enhanced peak where G4 sequences were located and reaction was promoted. Thus, the oxidized dopamine species were predominantly generated at the reaction centers and subsequent polymerization preferentially occurred close by, leading to polymer deposition in designated patterns. Due to the intrinsic adhesiveness of the biopolymer, polydopamine‐coated areas tend to aggregate and could even form higher‐ordered domains (Figure S13). The precise polymer formation with nanometer resolution was further demonstrated by eliminating the DNA template (Figure S14). After treating the mica‐deposited sample with hydrochloric acid, the DNA scaffold was degraded through hydrolysis, liberating the polydopamine nanostructures.

In order to achieve not only exact positioning of polydopamine on the DNA platform but also temporal control with light, the reaction mixture containing tube I was alternatingly exposed to visible light or kept in dark; the absorbance spectra were recorded at distinct time points in order to visualize the polymerization progress (Figure 2 A–C). The experiment commenced with a dark period of one hour and the light‐dependency of the reaction was revealed: In the absence of light, there was almost no formation of oxidized species and initiation was efficiently suppressed. The dopamine conversion was only triggered after one hour of irradiation, and early intermediates, e.g., dopaminochrome and oligomers, developed. During the ensuing dark period, the absorbance spectra marginally shifted; thus, polymerization was essentially intercepted due to a negligible concentration of radicals. Resuming irradiation caused significantly enhanced overall absorption, indicating that polymerization proceeds. The stepwise progression, as visualized by the relative absorbance increase, substantiates the temporal control through simple switching of the light source on and off (Figure 2 C). Furthermore, in addition to qualitative detection, the impact on polymer density and height was investigated via AFM (Figure 2 D,E). After one cycle of dark and irradiation phases, there was initial evidence of polydopamine formation. Beside the tubes whose height profile was determined by G4 secondary structures (approximately 3–4 nm in proportion to tube surface), some tubes increased in height due to polydopamine deposition. Extensive polymer coverage occurred upon a successive cycle of dark and irradiation periods, as seen by the dense rings in the center of the tubes, corresponding to an average height increase of up to 10 nm and a total height of 15 nm at these positions (Figure 2 E).

**Figure 2:**
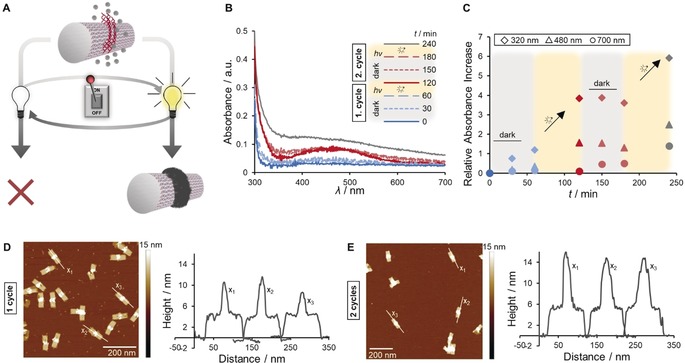
A) Temporal control over the polydopamine formation when the light is switched on and off (1 cycle consists of 1 h dark + 1 h light). B,C) UV/Vis spectroscopy indicates a light‐dependent, stepwise polydopamine formation process. D,E) The stepwise polymer growth becomes apparent in AFM topographic images: After 1 cycle, tubes show only a slight increase in height, whereas an approximately 10 nm thick polydopamine layer is present after an additional cycle.

DNA origami is emerging as a precise and functional nanomaterial for various biomedical and chemical applications. However, it would be highly desirable to circumvent the intrinsic stability issues and push the limits towards a robust but still versatile nanomaterial in various environments.^23^ Apart from nuclease‐mediated digestion, magnesium ions are crucial for DNA origami assembly, but their relatively high concentration (10–20 mm) can interfere with certain applications.^24^ Hence, polydopamine coating was anticipated as an suitable tool to contribute to stability. Repetitive spin filtering was used to transfer bare origami tubes and polydopamine‐decorated tube structures from storage buffer into virtually pure water, and these mixtures were incubated for several hours at elevated temperature (Figure 3). Topographical imaging of the nonmodified DNA tubes after treatment revealed a loss of integrity for almost all objects, ranging from small fragments through unfolded segments to intact parts of the original structure. In contrast, tubes bearing a centered ring of polydopamine seem to be less susceptible to variations in ionic environment. Apart from clusters of several tubes due to the adhesiveness of polydopamine, individually resolved tubes possess an intact architecture. Hence, when polydopamine is deposited on DNA origami, the structural integrity of DNA is maintained since its inherent susceptibility to lacking ions is reduced.

**Figure 3:**
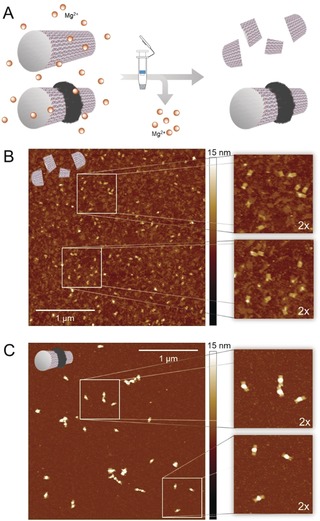
A) Spin‐filtering was used to transfer unmodified and polymer‐modified origami structures from Mg^2+^‐containing storage buffer into an ion‐free environment. Stability assay of unmodified (B) and polydopamine‐coated tubes (C) was conducted in pure water at 37 °C for 4 h; the structures were subsequently visualized by AFM, showing that polydopamine deposition enhanced the structural integrity.

In conclusion, we have reported the first phototriggered polydopamine polymerization on 3D DNA origami templates to create precise nanostructures. The system benefits from both the unique customization of the DNA origami methodology and the adjustability of utilizing visible light as an external stimulus to achieve unprecedented spatiotemporal control on the nanoscale. Various DNA origami templates were equipped with patterns of G‐quadruplex secondary structures, which—along with the embedded photosensitizer protoporphyrin IX—dictated the locally restricted formation of polydopamine. By suppressing the self‐polymerization of dopamine at slightly acidic pH and simply switching the light on and off, the characteristics, e.g., density and height of the polymer were tailored. Despite the sophisticated nature of the polydopamine‐decorated DNA origami objects, the setup was straightforward, and all materials were biologically derived. The combination of DNA origami technology and polymerizations initiated by visible light allowed the creation of precise photopatterned 3D nanostructures with dimensions far below 100 nm and much smaller than the wavelength of light. Furthermore, the deposition of polydopamine enhanced DNA origami stability, so that it can withstand even pure water conditions, broadening its application scope. Hence, the technique serves as an advanced toolkit for developing DNA‐based devices with nanometer resolution, which exhibit customized properties and stability features.

## Conflict of interest

The authors declare no conflict of interest.

## Supporting information

As a service to our authors and readers, this journal provides supporting information supplied by the authors. Such materials are peer reviewed and may be re‐organized for online delivery, but are not copy‐edited or typeset. Technical support issues arising from supporting information (other than missing files) should be addressed to the authors.

Supplementary
