# Supplementary material for: Photocontrolled Dopamine Polymerization on DNA Origami with Nanometer Resolution
Source: Angew Chem Int Ed Engl. 2019 Dec 27;59(15):6144–9. doi: 10.1002/anie.201911249 (PMC7186833; doi:10.1002/anie.201911249)
Supplement: Supplementary file 1 — Supplementary [file ANIE-59-6144-s001.pdf]

Angewandte Chemie
Eine Zeitschrift der Gesellschaft Deutscher Chemiker

Supporting Information

Photocontrolled Dopamine Polymerization on DNA Origami with Nanometer Resolution

Pia Winterwerber, Sean Harvey, David Y. W. Ng, and Tanja Weil**

anie_201911249_sm_miscellaneous_information.pdf

Table of Contents

Experimental Procedures **p. 3**

- Materials and Instruments
- DNA Origami Structures
- Polydopamine Formation on DNA Origami Structures
- Switch on – Switch off
- Reactive Oxygen Species Assay (ROS Assay)
- Binding Assay
- Template Degradation
- Stability Assay
- Agarose Gel Electrophoresis (AGE)
- Atomic Force Microscopy (AFM)

Results and Discussion **p. 5**

- Figure S2. AFM images of DNA origami templates before polymerization.
- Figure S3. Binding assay of protoporphyrin IX to G-quadruplex sequences.
- Figure S4. Binding specificity assay of protoporphyrin IX to different guanine-rich sequences.
- Figure S5. Proposed mechanism of polydopamine formation.
- Figure S6. Reactive oxygen assay (ROS) assay of protoporphyrin IX.
- Figure S7. Polymerization kinetics of dopamine at different pH values.
- Figure S8. Negative control of polydopamine formation in the dark.
- Figure S9. Role of the photosensitizer in the polymerization process.
- Figure S10. Investigation of the reactive oxygen species-mediated polymerization process.
- Figure S11. AFM imaging of the origami tubes after polymerization in the absence of oxygen.
- Figure S12. Agarose gel electrophoresis (AGE) of scaffold DNA, tubes before polymerization, and tubes after polymerization.
- Figure S13. Effects of polydopamine coating on structural behavior of the tubes.
- Figure S14. Degradation of the DNA template.
- Staple DNA Sequences

References **p. 15**

Experimental Procedures

Materials and Instruments

All solvents and chemicals were purchased from commercial sources and were used without further purification (Disodium ethylenediaminetetraacetic acid (Na_2EDTA), Sigma; Trizma® Base (TRIS), Sigma; polyethylene glycol 8000 (PEG_{8000}), Roth; protoporphyrin IX (PPIX), Sigma; dopamine hydrochloride, Sigma; Sephacryl S-400 HR, GE Healthcare; *N,N*-dimethyl-4-nitrosoaniline (RNO), Sigma; imidazole, Roth; SYBR gold, Thermo Fisher Scientific; DNA gel loading dye, Thermo Fisher Scientific). DNA staple strands, G-quadruplex extended staple strands, and folding strands were purchased from Sigma. M13mp18 plasmid DNA was synthesized according to the literature.[1] Annealing of scaffold DNA and the respective staple strands was performed on a Bio-Rad MyCycler™ Thermal Cycler. UV/Vis and fluorescence spectroscopy were conducted on a Spark® Multimode Microplate Reader by Tecan.

DNA Origami Structures

DNA origami tiles were prepared by mixing M13mp18 Scaffold DNA (1 equiv.), staple strands (8 equiv.) and G-quadruplex extended staple strands (8 equiv.) in origami buffer (1 mM Na_2EDTA, 5 mM NaCl, 5 mM TRIS, 12 mM $MgCl_2$ pH 8). To form tube structures, staple strands located at the long edges of the tile were replaced by folding strands (2 equiv.), respectively. Annealing was performed by running a program from 70 °C to 20 °C over 2 hours (0.5 °C/min to 35 °C, 1 °C/min to 20 °C) and the obtained DNA origami structures were purified by PEG precipitation.[2] Therefore, the PEG solution (15% PEG_{8000} (w/v), 5 mM TRIS, 1 mM Na_2EDTA, 505 mM NaCl) was added to the reaction solution at a volume ratio of 1:1 and centrifuged for 25 min at 12.5 rpm, room temperature (RT). The supernatant was removed and the resuspended pellet was precipitated by applying the PEG precipitation method for additional 2 times. Sample concentration was determined by Spark® 20M with Nanoquant plateTM. DNA origami structures were stored in origami buffer at 4 °C.

Polydopamine Formation on DNA Origami Structures

Standard polydopamine formation on DNA origami structures was carried out in a total reaction volume of 50 µL (or 100 µL, respectively) in a UV-star 384 well plate. Stock solutions of compounds were prepared in reaction buffer (10 mM BIS-TRIS, pH 6.5) at the following concentrations: protoporphyrin IX (PPIX) at 10 µM and dopamine hydrochloride at 0.5 M. A typical reaction was conducted as follows: DNA origami with G4-sequences (10 nM, final concentration) was incubated with PPIX (1.5 equiv., relative to the amount of G4-sequences) in reaction buffer for 30–60 min. Dopamine hydrochloride (10 mM, final concentration) was added and the plate was placed on a shaker (300 rpm), in a distance of approximately 1 cm to the LED array light source (cold white LED, 4600 K–9000 K, 3.0 mW/cm^2, purchased from Thorlabs, Inc., see Figure S1). After a predetermined irradiation time interval, polymerization was stopped by removing the light source and polydopamine-DNA objects were purified using size exclusion chromatography (200 µL Sephacryl S-400 HR, equilibrated with reaction buffer; centrifuge settings: 2 min, 0.8 g, RT).

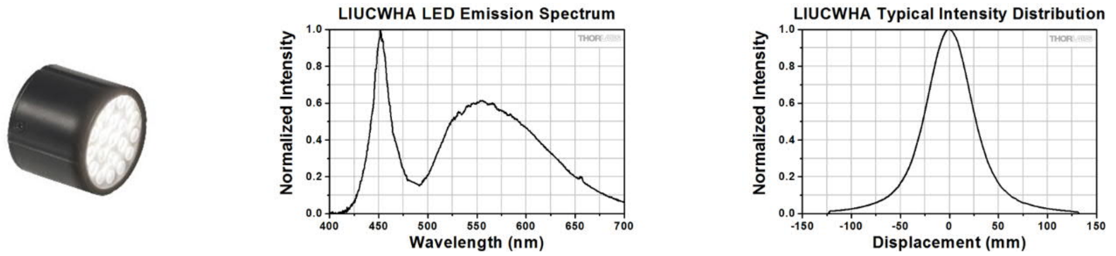

Figure S1. Cold white LED array employed in this study (left). Normalized emission spectrum of the cold white LED array (middle). Intensity distribution of the LED in the plane located 100 mm from the LED along the emission axis (right). Adapted from Manufacturer's specifications Thorlabs, Inc.

Polydopamine Formation in the Absence of Oxygen

In order to allow degassing of the system, the polymerization was conducted in 0.2 mL PCR tubes. The reaction mixture was prepared accordingly to the standard conditions. Imidazole stock solution in reaction buffer (0.5 M; adjusted to approx. pH 6.5) was applied at a final concentration of 100 mM. Oxygen was removed by gently degassing with Argon. To perform UV-Vis spectroscopy, the reaction solution was transferred to a 384 well plate, using the exact wells as the measurement before. The solutions were degassed after each measurement. After a reaction time of 180 min, work-up was conducted as described above.

Switch on – Switch off

Switch on – switch off studies were performed according to standard polydopamine formation protocol either on a shaker with 1 cm distance to the light source (irradiation phase) or in the Tecan plate reader (dark phase) for 1 h, respectively. Absorbance scans were conducted during the dark phase at t = 0, 15, 30, 45, 60 min. The reaction mixture was prepared in a 3.1-fold excess and split into 3 wells, whereas well 1, 2, and 3 were exposed to 1, 2, and 3 reaction cycles (1 h dark, 1 h irradiation), respectively. After each cycle, the corresponding reaction mixture was purified as described above for AFM imaging.

Reactive Oxygen Species Assay (ROS Assay)

Reactive oxygen species assay of PPIX was conducted in a total reaction volume of 150 µL in a 96 well plate. Wells were charged with PPIX (6.6 µM), *N,N*-dimethyl-4-nitrosoaniline (RNO, 70.7 µM) and imidazole (7.5 mM) in BIS-TRIS buffer (pH 6.5) and either exposed to visible light (cold white LED, 4600 K–9000 K, 3.0 mW/cm^2) or kept in dark. Absorbance scans from 300–500 nm were conducted at t = 0, 30, 90, 150, 210 min. The decrease in absorbance of RNO could be followed at 438 nm.

Binding Assay
Binding assay of PPIX and G4-sequences on DNA origami were performed in a total reaction volume of 16.6 µL in a black 384 well plate. PPIX (660 nM) in BIS-TRIS buffer (pH 6.5) was incubated with either DNA origami tile in origami buffer (10 nM) ("bound PPIX") or the same amount of blank origami buffer ("free PPIX"). Excitation wavelength was set at 420 nm and emission scans were recorded from 550–800 nm (bandwidth: 50 nm) every 5 min. Blank values were collected before PPIX addition.

Binding Specificity Assay
Binding specificity assay of PPIX and two different G-rich sequences (G4: 5'-TTTTGGGTAGGGCGGGTTGGG-3' and 12G: 5'-TTTTGAGTGCGAGTGCGAGTGCGAGTGC-3') was performed in a total reaction volume of 16.6 µL in a black 384 well plate. PPIX (660 nM) in BIS-TRIS buffer (pH 6.5) was incubated with DNA origami tubes in origami buffer (10 nM), bearing either a ring of G4 sequences ("Origami G4") or 12G sequences ("Origami 12G"). Excitation wavelength was set at 420 nm and emission scans were recorded from 600–800 nm (bandwidth: 20 nm) before and after addition of the origami structures.

Template Degradation
Polydopamine-coated DNA origami was added to a freshly cleaved mica substrate and incubated for 5 min to allow deposition. After selecting a suitable scanning area and taking an image, the scanner head was lifted in the z-direction. Few µL of 1 M HCl were added to the droplet of the origami buffer covering the mica surface, incubated for 5 min and the surface was approached again. Slight manual corrections in x- and y-direction were necessary to reimage previously observed areas.

Stability Assay
Desalting of samples was achieved by spin filtering with Amicon Ultra-0.5 mL Centrifugal Filters (MWCO 100K). 1 µL of precursor DNA origami (50 nM) or 5 µL of polydopamine-coated DNA origami (approx. 10 nM) were diluted with Milli-Q water to a total volume of 100 µL and centrifuged for 20 min at 12.5 g, RT. The concentrate volume of 15 µL was then refilled up to 100 µL with Milli-Q water, centrifuged, and the procedure was repeated two more times. After 1:1 (v/v) dilution with Milli-Q water, samples were incubated at 37 °C for 4 h and applied to mica substrate for AFM imaging. Final concentration of magnesium ions was calculated as 0.2 µM, based on a dead volume after spin filtering of 15 µL.

Agarose Gel Electrophoresis (AGE)
Agarose gel electrophoresis was performed on 1% agarose gels based on 0.5 × Tris-Borate-EDTA (TBE, diluted from 10 × TBE buffer concentrate from Sigma-Aldrich), equipped with 8 wells. The gels were run on ReadySub-Cell GT Cells from Bio-Rad using 0.5 × TBE buffer as the running buffer. DNA Gel Loading dye (6 ×) was used for sample preparation (approximately 50 fmol origami) with a total volume of 6 µL. Electrophoresis was conducted at 90 V for 90 minutes on ice. Gels were subsequently stained with SYBR Gold in 50 mL 0.5 × TBE buffer for 30 min and image was taken with G:BOX Chemi Gel Doc System from Syngene.

Atomic Force Microscopy (AFM)
Atomic force microscopy was conducted in liquid state with a Bruker Dimension FastScan Bio^{TM} atomic force microscope, which was operated in PeakForce mode. AFM probes with a nominal spring constant of 0.25 Nm^{-1} (FastScan-D, Bruker) were used. Sample solution (30 µL, 1-2 nM in origami buffer) was added onto a freshly cleaved mica substrate (circular, 15 mm) and incubated for 5 min to allow deposition of the origami structures. Remaining solution was removed and 300 µL origami buffer was applied onto the mica surface, forming a droplet for measuring in liquid. Samples were scanned with scan rates between 1 and 2 Hz. Images were processed with NanoScope Analysis 1.8.

Results and Discussion

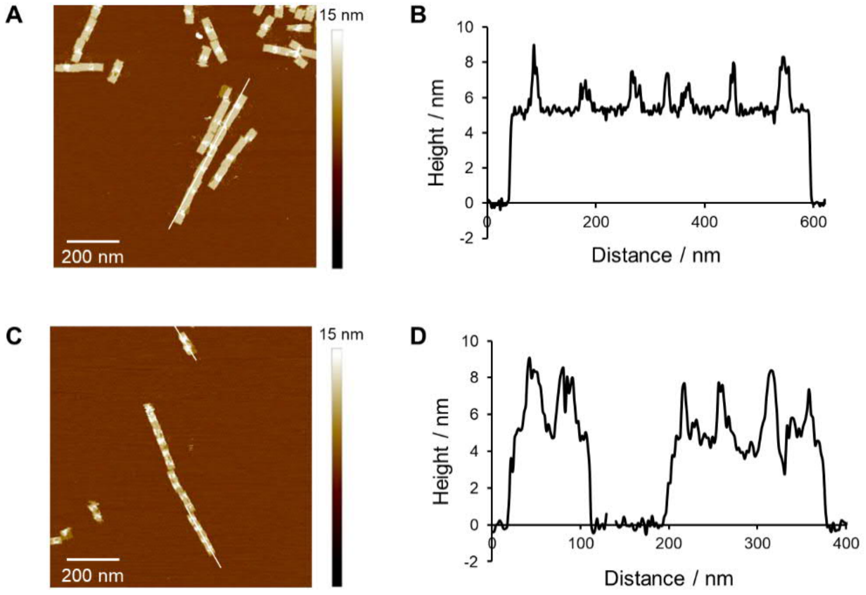

Figure S2. AFM characterization of DNA origami templates before polymerization. AFM topographic images of (A) a tube with 1 centered line of G-quadruplex (G4) sequences (tube I) and the corresponding height profile (B), and (C) a tube with 2 lines of G4 sequences (tube II) and the corresponding height profile (D).

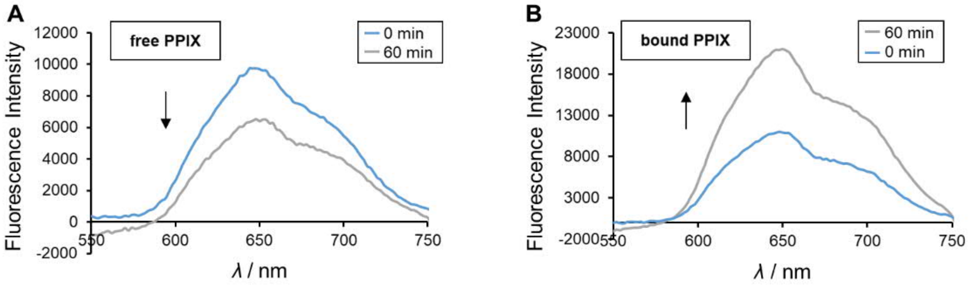

Figure S3. Binding assay of protoporphyrin IX to G-quadruplex sequences. Fluorescence intensity of protoporphyrin IX (PPIX) as (A) “free PPIX” and (B) “PPIX bound” to G-quadruplex sequences within the DNA origami framework. Upon binding, fluorescence significantly increases over time (60 min), whereas fluorescence of free PPIX in solution decreases. Spectra are blank-corrected.

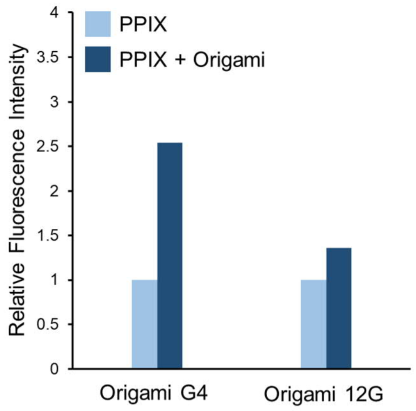

Figure S4. Binding specificity assay of protoporphyrin IX to different guanine-rich sequences. Upon addition of origami tubes bearing either G4 sequences ("Origami G4") or origami tubes with an alternately organized G-rich sequence ("Origami 12G"), only the fluorescence of the Origami G4-treated photosensitizer significantly increases, demonstrating a specific binding event.

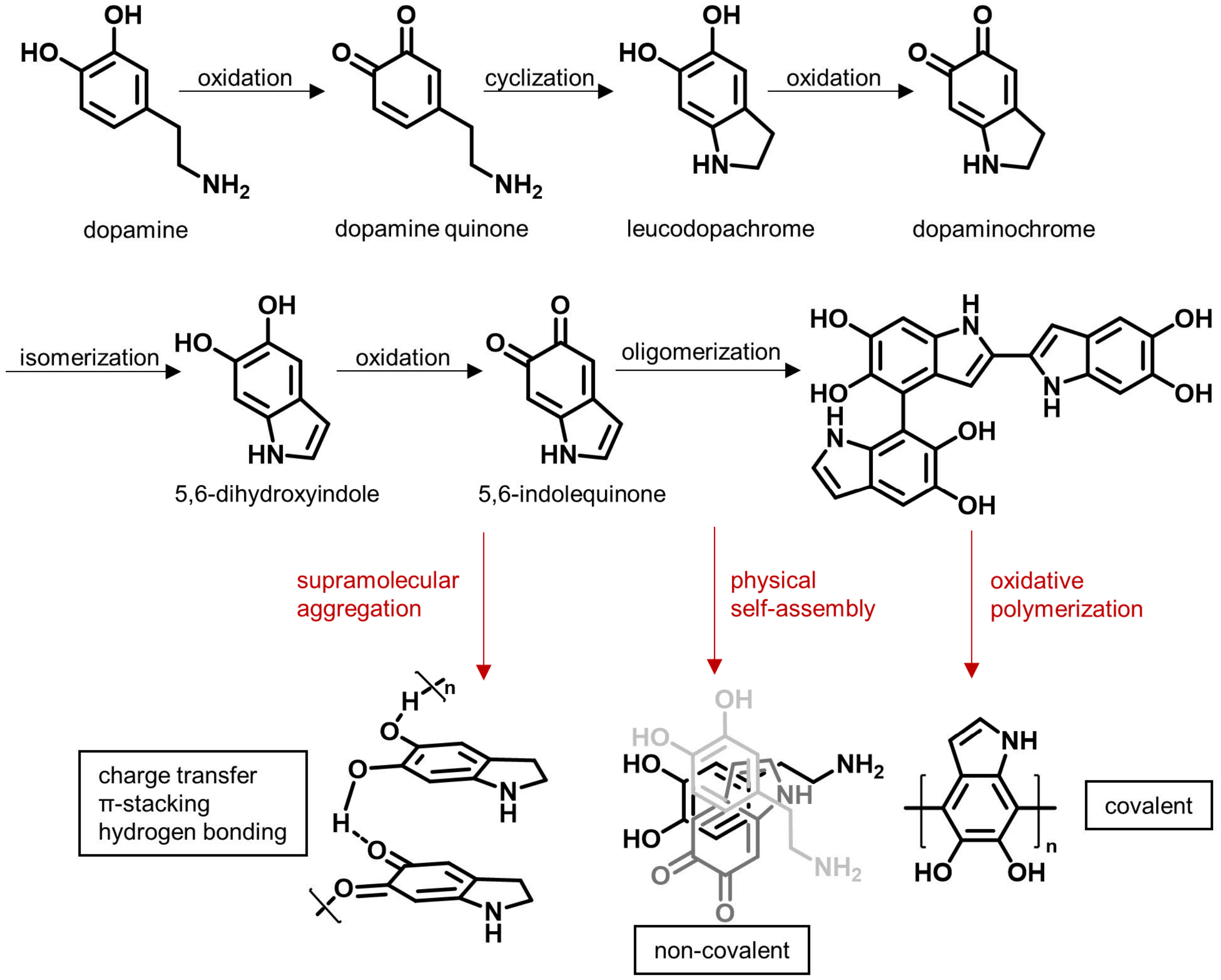

Figure S5. Proposed mechanism of polydopamine formation. After several oxidation steps, cyclization and isomerization, different pathways are suggested to contribute to the complex and many-faceted structure of polydopamine. Apart from covalent interactions generating oligomers and polymers, physical self-assembly and supramolecular aggregation based on charge transfer, *π*-stacking and hydrogen bonding are determining the polymer's structure. For reason of clarity and simplicity, only the main representative examples are depicted.[3]

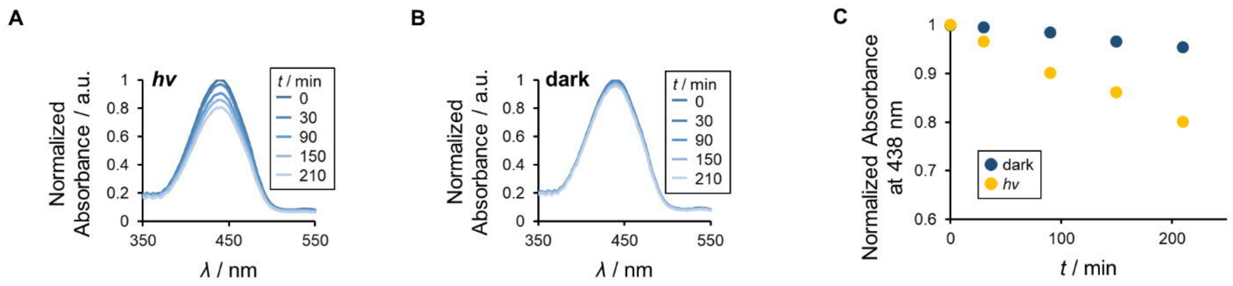

Figure S6. Reactive oxygen species (ROS) assay of protoporphyrin IX (PPIX). Singlet oxygen generated by PPIX upon irradiation with visible light reacts with imidazole and bleaches *N,N*-dimethyl-4-nitrosoaniline (RNO) through oxidation (A), while no reaction occurs in the absence of light (B). Absorbance of RNO was followed at 438 nm (C).

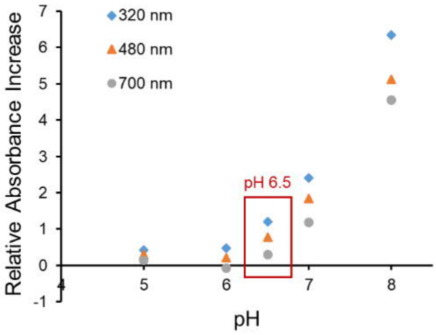

Figure S7. Polymerization kinetics of dopamine at different pH values. For pH studies, the following buffers were employed: pH 5: acetate buffer 10 mM, pH 6 and pH 7: phosphate buffer 10 mM, pH 6.5: BIS-TRIS buffer 10 mM, and pH 8: TRIS buffer 5 mM. Data collected at t = 120 min.

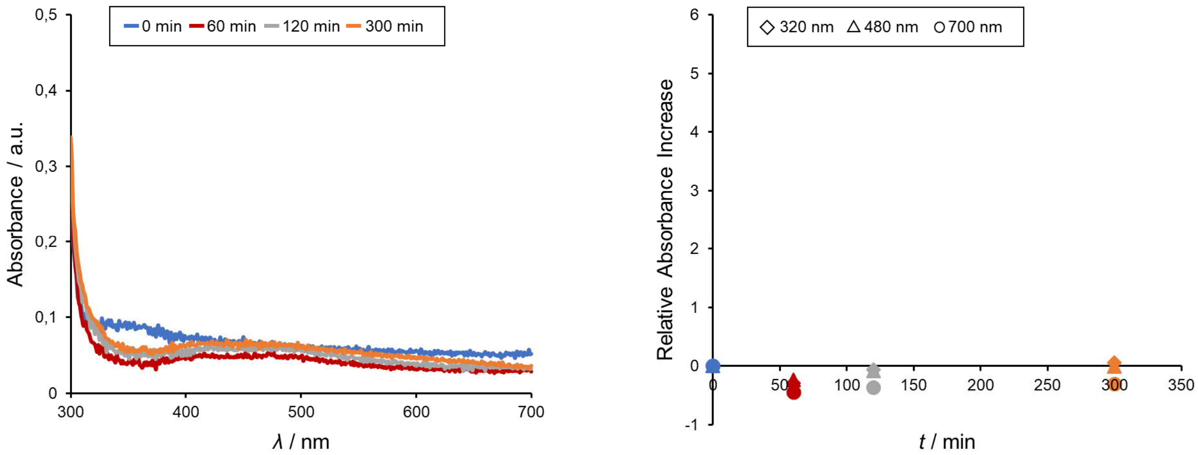

Figure S8. Negative control of polydopamine formation in the dark. UV/Vis spectroscopy of polydopamine formation on DNA origami tiles in BIS-TRIS buffer at pH 6.5 in the absence of light. Neither dopaminochrome, nor oligomer or polymer formation could be detected under these conditions.

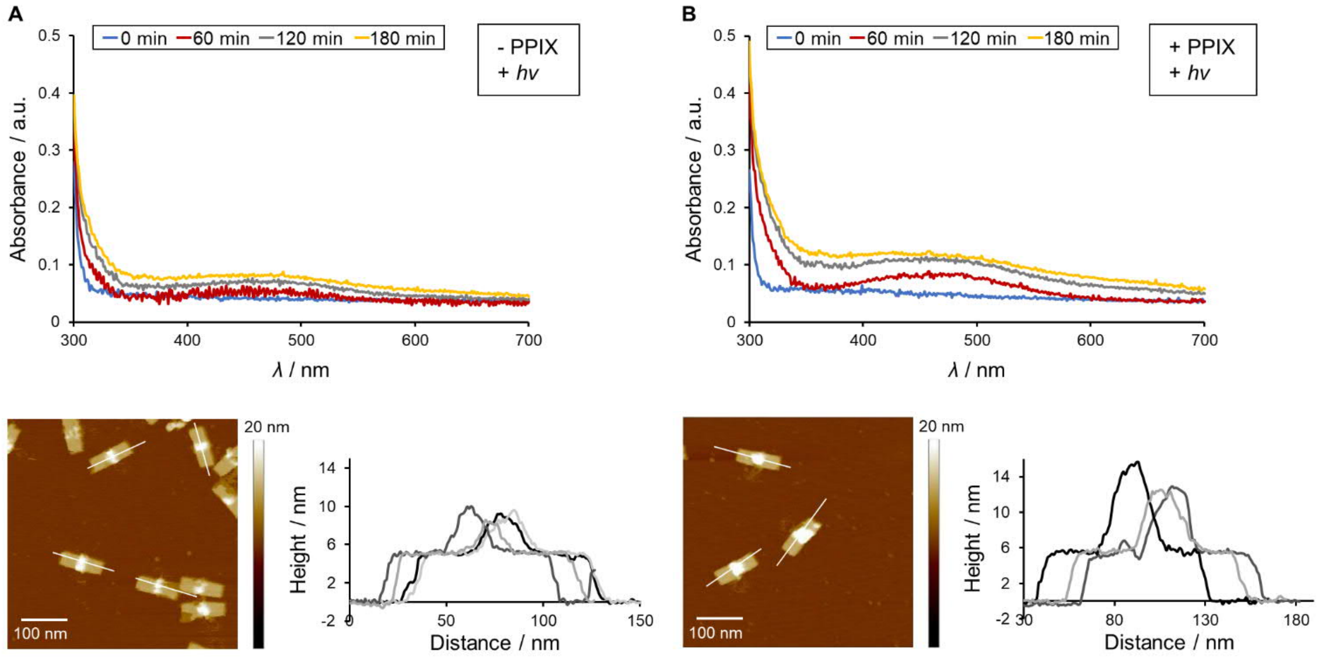

Figure S9. **Role of the photosensitizer in the polymerization process.** (A) Without protoporphyrin IX (PPIX), no polymerization takes place upon the irradiation with visible light, as visualized by UV/Vis spectroscopy and AFM. (B) Upon the embedding of PPIX and illumination, polydopamine formation is observed.

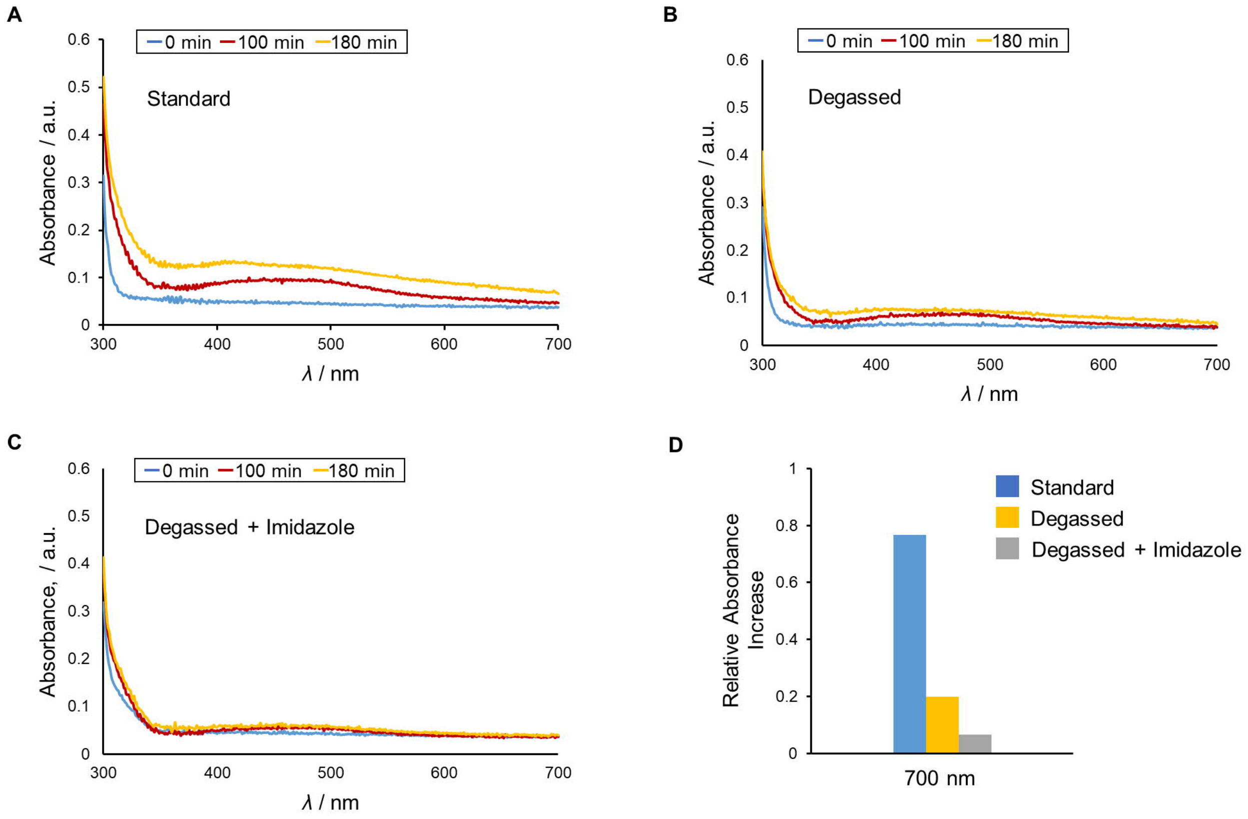

Figure S10. Investigation of the reactive oxygen species-mediated polymerization process under (A) standard conditions, (B) in the absence oxygen and (C) in the absence of oxygen but in the presence of imidazole as a scavenger, visualized by UV-Vis spectroscopy. Thereby, the formation of polydopamine is significantly suppressed (D).

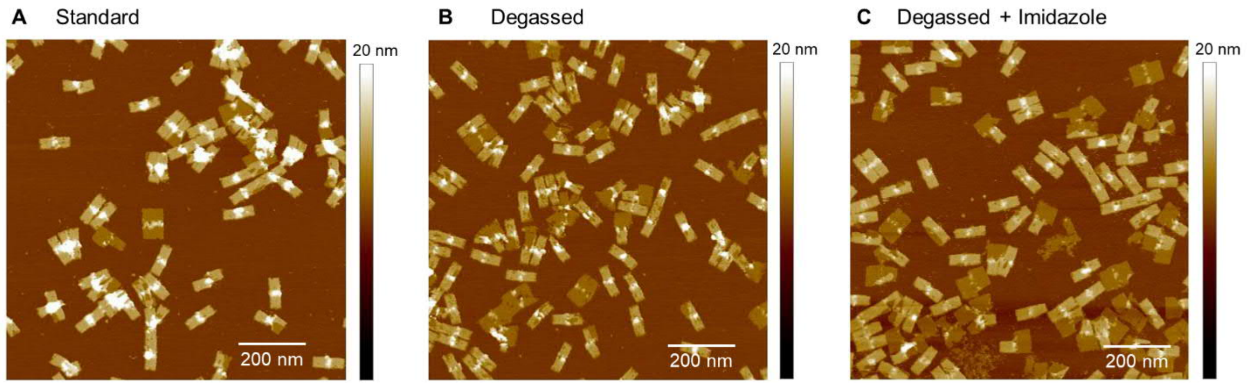

Figure S11. AFM imaging of the origami tubes after reaction under (A) standard, (B) degassed and (C) degassed and imidazole-added conditions also show a suppression of polydopamine formation in the absence of oxygen.

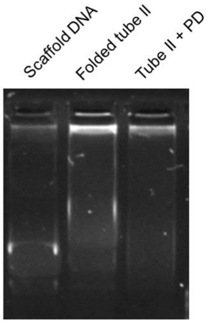

Figure S12. Agarose gel electrophoresis (AGE) of scaffold DNA, tubes before polymerization, and tubes after polymerization. Lanes were charged with scaffold DNA, folded origami tube II before polymerization, and polydopamine-coated tube II after polymerization. A shift in molecular weight upon folding is observed. Upon polydopamine-coating, migration of the polymer-DNA hybrid materials is completely suppressed. For reason of clarity, excess staples in the low molecular weight region are not shown.

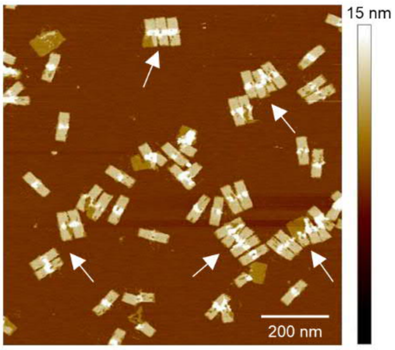

Figure S13. Effects of polydopamine coating on structural behavior of the tubes. Due to the adhesiveness of polydopamine, tubes stick to each other, forming higher-ordered domains.

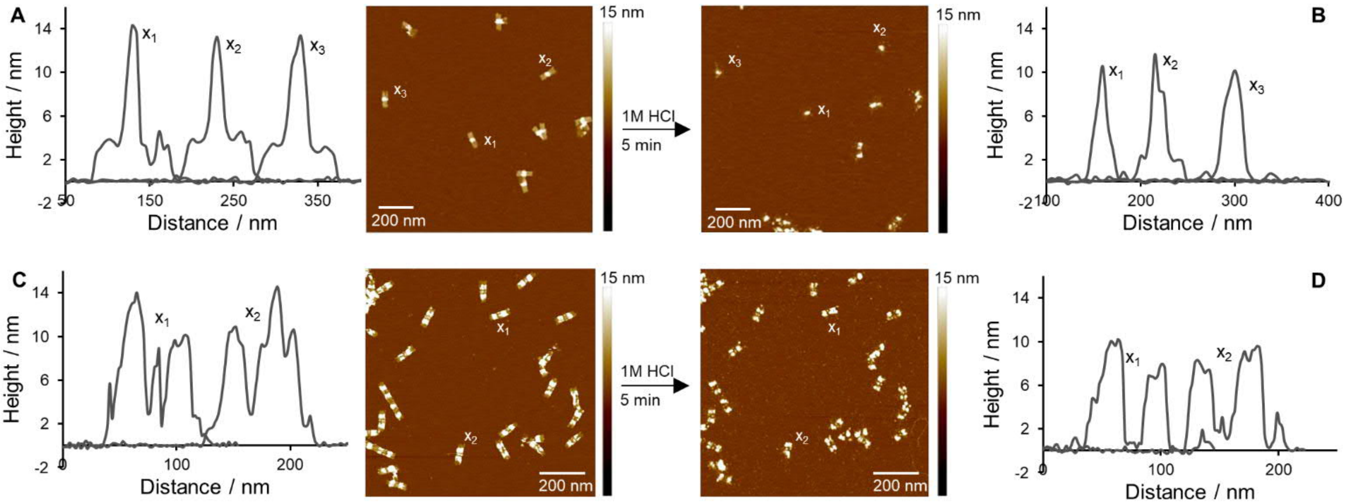

Figure S14. Degradation of the DNA template. Treatment of the polydopamine-coated origami tube I (A) and tube II (C) with hydrochloric acid during AFM measurements. Upon the addition of acid, the DNA template is degraded, liberating the polydopamine structures (B+D).

Staple DNA Sequences

DNA origami structures were synthesized together with M13mp18 Scaffold DNA according to the following protocol. For G-quadruplex extended staple strands (G4 staple strands), the sequence of the corresponding staple strand is extended at the 3’ end by 5’-TTTTGGGTAGGGCGGGTTGGG-3’.

Tile: G4 strands on positions 29, 30, 31–40, 41–50, 53–60, 61–70, 71–74; staple strands on remaining positions

Tube I: G4 strands on positions 2–10, 11–20, 21–24, 26, 112–120, 121–130, 131; folding strands on positions 1, 25, 27, 28, 51, 52, 75, 76, 99, 100, 111, 132, 133, 156, 157, 180, 181, 204, 205, 216; staple strands on remaining positons

Tube II: G4 strands on positions 29, 30, 31–40, 41–50, 53–60, 61–70, 71–74, 134–140, 141–150, 151–155, 158–160, 161–170, 171–179; folding strands on positions 1, 25, 27, 28, 51, 52, 75, 76, 99, 100, 111, 132, 133, 156, 157, 180, 181, 204, 205, 216; staple strands on remaining positons

Table S1. Sequences of staple strands and folding strands.

Position	Sequence 5’ → 3’
1	CAAGCCCAATAGGAACCCATGTACAAACAGTT
2	AATGCCCCGTAACAGTGCCCGTATCTCCCTCA
3	TGCCTTGACTGCCTATTTCGGAACAGGGATAG
4	GAGCCGCCCCACCACCGGAACCGCGACGGAAA
5	AACCAGAGACCCTCAGAACCGCCAGGGGTCAG
6	TTATTCATAGGGAAGGTAAATATTCATTCAGT
7	CATAACCCGAGGCATAGTAAGAGCTTTTTAAG
8	ATTGAGGGTAAAGGTGAATTATCAATCACCGG
9	AAAAGTAATATCTTACCGAAGCCCTTCCAGAG
10	GCAATAGCGCAGATAGCCGAACAATTCAACCG
11	CCTAATTTACGCTAACGAGCGTCTAATCAATA
12	TCTTACCAGCCAGTTACAAAATAAATGAAATA
13	ATCGGCTGCGAGCATGTAGAAACCTATCATAT
14	CTAATTTATCTTTCCTTATCATTCATCCTGAA
15	GCGTTATAGAAAAAGCCTGTTTAGAAGGCCGG
16	GCTCATTTTCGCATTAAATTTTTGAGCTTAGA
17	AATTACTACAAATTCTTACCAGTAATCCCATC
18	TTAAGACGTTGAAAACATAGCGATAACAGTAC
19	TAGAATCCCTGAGAAGAGTCAATAGGAATCAT
20	CTTTTACACAGATGAATATACAGTAAACAATT
21	TTTAACGTTCGGGAGAAACAATAATTTTCCCT
22	CGACAACTAAGTATTAGACTTTACAATACCGA
23	GGATTTAGCGTATTAAATCCTTTGTTTTCAGG
24	ACGAACCAAAACATCGCCATTAAATGGTGGTT
25	GAACGTGGCGAGAAAGGAAGGGAACAAACTAT
26	TAGCCCTACCAGCAGAAGATAAAAACATTTGA
27	CGGCCTTGCTGGTAATATCCAGAACGAACTGA
28	CTCAGAGCCACCACCCTCATTTTCCTATTATT
29	CTGAAACAGGTAATAAGTTTTAACCCCTCAGA
30	AGTGTACTTGAAAGTATTAAGAGGCCGCCACC
31	GCCACCACTCTTTTCATAATCAAACCGTCACC
32	GTTTGCCACCTCAGAGCCGCCACCGATACAGG
33	GACTTGAGAGACAAAAGGGCGACAAGTTACCA
34	AGCGCCAACCATTTGGGAATTAGATTATTAGC
35	GAAGGAAAATAAGAGCAAGAAACAACAGCCAT
36	GCCCAATACCGAGGAAACGCAATAGGTTTACC
37	ATTATTTAACCCAGCTACAATTTTCAAGAACG
38	TATTTTGCTCCCAATCCAAATAAGTGAGTTAA
39	GGTATTAAGAACAAGAAAAATAATTAAAGCCA
40	TAAGTCCTACCAAGTACCGCACTCTTAGTTGC
41	ACGCTCAAAATAAGAATAAACACCGTGAATTT
42	AGGCGTTACAGTAGGGCTTAATTGACAATAGA
43	ATCAAAATCGTCGCTATTAATTAACGGATTCG

44	CTGTAAATCATAGGTCTGAGAGACGATAAATA
45	CCTGATTGAAAGAAATTGCGTAGACCCGAACG
46	ACAGAAATCTTTGAATACCAAGTTCCTTGCTT
47	TTATTAATGCCGTCAATAGATAATCAGAGGTG
48	AGATTAGATTTAAAAGTTTGAGTACACGTAAA
49	AGGCGGTCATTAGTCTTTAATGCGCAATATTA
50	GAATGGCTAGTATTAACACCGCCTCAACTAAT
51	CCGCCAGCCATTGCAACAGGAAAAATATTTTT
52	CCCTCAGAACCGCCACCCTCAGAACTGAGACT
53	CCTCAAGAATACATGGCTTTTGATAGAACCAC
54	TAAGCGTCGAAGGATTAGGATTAGTACCGCCA
55	CACCAGAGTTCGGTCATAGCCCCCGCCAGCAA
56	TCGGCATTCCGCCGCCAGCATTGACGTTCCAG
57	AATCACCAAATAGAAAATTCATATATAACGGA
58	TCACAATCGTAGCACCATTACCATCGTTTTCA
59	ATACCCAAGATAACCCACAAGAATAAACGATT
60	ATCAGAGAAAGAACTGGCATGATTTTATTTTG
61	TTTTGTTTAAGCCTTAAATCAAGAATCGAGAA
62	AGGTTTTGAACGTCAAAAATGAAAGCGCTAAT
63	CAAGCAAGACGCGCCTGTTTATCAAGAATCGC
64	AATGCAGACCGTTTTTATTTTCATCTTGCGGG
65	CATATTTAGAAATACCGACCGTGTTACCTTTT
66	AATGGTTTACAACGCCAACATGTAGTTCAGCT
67	TAACCTCCATATGTGAGTGAATAAACAAAATC
68	AAATCAATGGCTTAGGTTGGGTTACTAAATTT
69	GCGCAGAGATATCAAAATTATTTGACATTATC
70	AACCTACCGCGAATTATTCATTTCCAGTACAT
71	ATTTTGCGTCTTTAGGAGCACTAAGCAACAGT
72	CTAAAATAGAACAAAGAAACCACCAGGGTTAG
73	GCCACGCTATACGTGGCACAGACAACGCTCAT
74	GCGTAAGAGAGAGCCAGCAGCAAAAAGGTTAT
75	GGAAATACCTACATTTTGACGCTCACCTGAAA
76	TATCACCGTACTCAGGAGGTTTAGCGGGGTTT
77	TGCTCAGTCAGTCTCTGAATTTACCAGGAGGT
78	GGAAAGCGACCAGGCGGATAAGTGAATAGGTG
79	TGAGGCAGGCGTCAGACTGTAGCGTAGCAAGG
80	TGCCTTTAGTCAGACGATTGGCCTGCCAGAAT
81	CCGGAAACACACCACGGAATAAGTAAGACTCC
82	ACGCAAAGGTCACCAATGAAACCAATCAAGTT
83	TTATTACGGTCAGAGGGTAATTGAATAGCAGC
84	TGAACAAACAGTATGTTAGCAAACTAAAAGAA
85	CTTTACAGTTAGCGAACCTCCCGACGTAGGAA
86	GAGGCGTTAGAGAATAACATAAAAGAACACCC
87	TCATTACCCGACAATAAACAACATATTTAGGC
88	CCAGACGAGCGCCCAATAGCAAGCAAGAACGC
89	AGAGGCATAATTTCATCTTCTGACTATAACTA
90	TTTTAGTTTTTCGAGCCAGTAATAAATTCTGT
91	TATGTAAACCTTTTTTAATGGAAAAATTACCT
92	TTGAATTATGCTGATGCAAATCCACAAATATA
93	GAGCAAAAACTTCTGAATAATGGAAGAAGGAG
94	TGGATTATGAAGATGATGAAACAAAATTTCAT
95	CGGAATTATTGAAAGGAATTGAGGTGAAAAAT
96	ATCAACAGTCATCATATTCCTGATTGATTGTT
97	CTAAAGCAAGATAGAACCCTTCTGAATCGTCT
98	GCCAACAGTCACCTTGCTGAACCTGTTGGCAA
99	GAAATGGATTATTTACATTGGCAGACATTCTG
100	TTTTTATAAGTATAGCCCGGCCGTCGAG
101	AGGGTTGATTTTATAAATCCTCATTAAATGATATTC
102	ACAAACAATTTTAATCAGTAGCGACAGATCGATAGC

103	AGCACCGTTTTTTAAAGGTGGCAACATAGTAGAAAA
104	TACATACATTTTGACGGGAGAATTAACTACAGGGAA
105	GCGCATTATTTTGCTTATCCGGTATTCTAAATCAGA
106	TATAGAAGTTTTCGACAAAAGGTAAAGTAGAGAATA
107	TAAAGTACTTTTCGCGAGAAAACTTTTTATCGCAAG
108	ACAAAGAATTTTATTAATTACATTTAACACATCAAG
109	AAAACAAATTTTTTCATCAATATAATCCTATCAGAT
110	GATGGCAATTTTAATCAATATCTGGTCACAAATATC
111	AAACCCTCTTTTACCAGTAATAAAAGGGATTCACCAGTCACACGTTTT
112	CCGAAATCCGAAAATCCTGTTTGAAGCCGGAA
113	CCAGCAGGGGCAAAATCCCTTATAAAGCCGGC
114	GCATAAAGTTCCACACAACATACGAAGCGCCA
115	GCTCACAATGTAAAGCCTGGGGTGGGTTTGCC
116	TTCGCCATTGCCGGAAACCAGGCATTAAATCA
117	GCTTCTGGTCAGGCTGCGCAACTGTGTTATCC
118	GTTAAAATTTTAACCAATAGGAACCCGGCACC
119	AGACAGTCATTCAAAAGGGTGAGAAGCTATAT
120	AGGTAAAGAAATCACCATCAATATAATATTTT
121	TTTCATTTGGTCAATAACCTGTTTATATCGCG
122	TCGCAAATGGGGCGCGAGCTGAAATAATGTGT
123	TTTTAATTGCCCGAAAGACTTCAAAACACTAT
124	AAGAGGAACGAGCTTCAAAGCGAAGATACATT
125	GGAATTACTCGTTTACCAGACGACAAAAGATT
126	GAATAAGGACGTAACAAAGCTGCTCTAAAACA
127	CCAAATCACTTGCCCTGACGAGAACGCCAAAA
128	CTCATCTTGAGGCAAAAGAATACAGTGAATTT
129	AAACGAAATGACCCCCAGCGATTATTCATTAC
130	CTTAAACATCAGCTTGCTTTCGAGCGTAACAC
131	TCGGTTTAGCTTGATACCGATAGTCCAACCTA
132	TGAGTTTCGTCACCAGTACAAACTTAATTGTA
133	CCCCGATTTAGAGCTTGACGGGGAAATCAAAA
134	GAATAGCCGCAAGCGGTCCACGCTCCTAATGA
135	GAGTTGCACGAGATAGGGTTGAGTAAGGGAGC
136	GTGAGCTAGTTTCCTGTGTGAAATTTGGGAAG
137	TCATAGCTACTCACATTAATTGCGCCCTGAGA
138	GGCGATCGCACTCCAGCCAGCTTTGCCATCAA
139	GAAGATCGGTGCGGGCCTCTTCGCAATCATGG
140	AAATAATTTTAAATTGTAAACGTTGATATTCA
141	GCAAATATCGCGTCTGGCCTTCCTGGCCTCAG
142	ACCGTTCTAAATGCAATGCCTGAGAGGTGGCA
143	TATATTTTAGCTGATAAATTAATGTTGTATAA
144	TCAATTCTTTTAGTTTGACCATTACCAGACCG
145	CGAGTAGAACTAATAGTAGTAGCAAACCCTCA
146	GAAGCAAAAAAGCGGATTGCATCAGATAAAAA
147	TCAGAAGCCTCCAACAGGTCAGGATCTGCGAA
148	CCAAAATATAATGCAGATACATAAACACCAGA
149	CATTCAACGCGAGAGGCTTTTGCATATTATAG
150	ACGAGTAGTGACAAGAACCGGATATACCAAGC
151	AGTAATCTTAAATTGGGCTTGAGAGAATACCA
152	GCGAAACATGCCACTACGAAGGCATGCGCCGA
153	ATACGTAAAAGTACAACGGAGATTTCATCAAG
154	CAATGACACTCCAAAAGGAGCCTTACAACGCC
155	AAAAAAGGACAACCATCGCCCACGCGGGTAAA
156	TGTAGCATTCCACAGACAGCCCTCATCTCCAA
157	GTAAAGCACTAAATCGGAACCCTAGTTGTTCC
158	AGTTTGGAGCCCTTCACCGCCTGGTTGCGCTC
159	AGCTGATTACAAGAGTCCACTATTGAGGTGCC
160	ACTGCCCGCCGAGCTCGAATTCGTTATTACGC
161	CCCGGGTACTTTCCAGTCGGGAAACGGGCAAC

162	CAGCTGGCGGACGACGACAGTATCGTAGCCAG
163	GTTTGAGGGAAAGGGGGATGTGCTAGAGGATC
164	CTTTCATCCCCAAAAACAGGAAGACCGGAGAG
165	AGAAAAGCAACATTAAATGTGAGCATCTGCCA
166	GGTAGCTAGGATAAAAATTTTTAGTTAACATC
167	CAACGCAATTTTTGAGAGATCTACTGATAATC
168	CAATAAATACAGTTGATTCCCAATTTAGAGAG
169	TCCATATACATACAGGCAAGGCAACTTTATTT
170	TACCTTTAAGGTCTTTACCCTGACAAAGAAGT
171	CAAAAATCATTGCTCCTTTTGATAAGTTTCAT
172	TTTGCCAGATCAGTTGAGATTTAGTGGTTTAA
173	AAAGATTCAGGGGGTAATAGTAAACCATAAAT
174	TTTCAACTATAGGCTGGCTGACCTTGTATCAT
175	CCAGGCGCTTAATCATTGTGAATTACAGGTAG
176	CGCCTGATGGAAGTTTCCATTAAACATAACCG
177	TTTCATGAAAATTGTGTCGAAATCTGTACAGA
178	ATATATTCTTTTTTCACGTTGAAAATAGTTAG
179	AATAATAAGGTCGCTGAGGCTTGCAAAGACTT
180	CGTAACGATCTAAAGTTTTGTCGTGAATTGCG
181	ACCCAAATCAAGTTTTTTGGGGTCAAAGAACG
182	TGGACTCCCTTTTCACCAGTGAGACCTGTCGT
183	TGGTTTTTAACGTCAAAGGGCGAAGAACCATC
184	GCCAGCTGCCTGCAGGTCGACTCTGCAAGGCG
185	CTTGCATGCATTAATGAATCGGCCCGCCAGGG
186	ATTAAGTTCGCATCGTAACCGTGCGAGTAACA
187	TAGATGGGGGGTAACGCCAGGGTTGTGCCAAG
188	ACCCGTCGTCATATGTACCCCGGTAAAGGCTA
189	CATGTCAAGATTCTCCGTGGGAACCGTTGGTG
190	TCAGGTCACTTTTGCGGGAGAAGCAGAATTAG
191	CTGTAATATTGCCTGAGAGTCTGGAAAACTAG
192	CAAAATTAAAGTACGGTGTCTGGAAGAGGTCA
193	TGCAACTAAGCAATAAAGCCTCAGTTATGACC
194	TTTTTGCGCAGAAAACGAGAATGAATGTTTAG
195	AAACAGTTGATGGCTTAGAGCTTATTTAAATA
196	ACTGGATAACGGAACAACATTATTACCTTATG
197	ACGAACTAGCGTCCAATACTGCGGAATGCTTT
198	CGATTTTAGAGGACAGATGAACGGCGCGACCT
199	CTTTGAAAAGAACTGGCTCATTATTTAATAAA
200	GCTCCATGAGAGGCTTTGAGGACTAGGGAGTT
201	ACGGCTACTTACTTAGCCGGAACGCTGACCAA
202	AAAGGCCGAAAGGAACAACTAAAGCTTTCCAG
203	GAGAATAGCTTTTGCGGGATCGTCGGGTAGCA
204	ACGTTAGTAAATGAATTTTCTGTAAGCGGAGT
205	TTTTCGATGGCCCACTACGTAAACCGTC
206	TATCAGGGTTTTCGGTTTGCGTATTGGGAACGCGCG
207	GGGAGAGGTTTTTGTAAAACGACGGCCATTCCCAGT
208	CACGACGTTTTTGTAATGGGATAGGTCAAAACGGCG
209	GATTGACCTTTTGATGAACGGTAATCGTAGCAAACA
210	AGAGAATCTTTTGGTTGTACCAAAAACAAGCATAAA
211	GCTAAATCTTTTCTGTAGCTCAACATGTATTGCTGA
212	ATATAATGTTTTCATTGAATCCCCCTCAAATCGTCA
213	TAAATATTTTTTGGAAGAAAAATCTACGACCAGTCA
214	GGACGTTGTTTTTCATAAGGGAACCGAAAGGCGCAG
215	ACGGTCAATTTTGACAGCATCGGAACGAACCCTCAG
216	CAGCGAAAATTTTACTTTCAACAGTTTCTGGGATTTTGCTAAACTTTT
217	AACATCACTTGCCTGAGTAGAAGAACT
218	TGTAGCAATACTTCTTTGATTAGTAAT
219	AGTCTGTCCATCACGCAAATTAACCGT
220	ATAATCAGTGAGGCCACCGAGTAAAAG

221	ACGCCAGAATCCTGAGAAGTGTTTTT
222	TTAAAGGGATTTTAGACAGGAACGGT
223	AGAGCGGGAGCTAAACAGGAGGCCGA
224	TATAACGTGCTTTCCTCGTTAGAATC
225	GTACTATGGTTGCTTTGACGAGCACG
226	GCGCTTAATGCGCCGCTACAGGGCGC
F1	CGGCCTTGATAGGAACCCATGTACAAACAGTT
F25	TGAGTTTCCGAGAAAGGAAGGGAACAAACTAT
F27	CAAGCCCACTGGTAATATCCAGAACGAACTGA
F28	CCGCCAGCCACCACCCTCATTTTCCTATTATT
F51	CTCAGAGCCATTGCAACAGGAAAAATATTTTT
F52	GGAAATACACCGCCACCCTCAGAACTGAGACT
F75	CCCTCAGACTACATTTTGACGCTCACCTGAAA
F76	GAAATGGATACTCAGGAGGTTTAGCGGGGTTT
F99	TATCACCGTTATTTACATTGGCAGACATTCTG
F132	GAACGTGGGTCACCAGTACAAACTTAATTGTA
F133	TGTAGCATTAGAGCTTGACGGGGAAATCAAAA
F156	CCCCGATTTCCACAGACAGCCCTCATCTCCAA
F157	CGTAACGACTAAATCGGAACCCTAGTTGTTCC
F180	GTAAAGCATCTAAAGTTTTGTCGTGAATTGCG
F181	ACGTTAGTCAAGTTTTTTGGGGTCAAAGAACG
F204	ACCCAAATAAATGAATTTTCTGTAAGCGGAGT
F100	GTCACACGTTTTTATAAGTATAGCCCGGCCGTCGAG
F205	TGCTAAACTTTTCGATGGCCCACTACGTAAACCGTC
N-111	AAACCCTCTTTTACCAGTAATAAAAGGGATTCACCA
N-216	CAGCGAAATTTTAACTTTCAACAGTTTCTGGGATTT

References

[1] G. Bellot, M. A. McClintock, J. J. Chou, W. M. Shih, *Nat. Protoc.* **2013**, *8*, 755-770.

[2] E. Stahl, T. G. Martin, F. Praetorius, H. Dietz, *Angew. Chem. Int. Ed.* **2014**, *53*, 12735-12740.

[3] a) H. Lee, S. M. Dellatore, W. M. Miller, P. B. Messersmith, *Science* **2007**, *318*, 426-430; b) D. R. Dreyer, D. J. Miller, B. D. Freeman, D. R. Paul, C. W. Bielawski, *Langmuir* **2012**, *28*, 6428-6435; c) S. Hong, Y. S. Na, S. Choi, I. T. Song, W. Y. Kim, H. Lee, *Adv. Funct. Mater.* **2012**, *22*, 4711-4717.

Author Contributions

P. W. conducted all the experiments and analysis assisted by S. H. on the experimental design. The project was supervised by D.Y.W.N. and T.W. All authors contributed to writing the manuscript.
